# The Role of Spirituality and Religion in Improving Quality of Life and Coping Mechanisms in Cancer Patients

**DOI:** 10.3390/healthcare12232349

**Published:** 2024-11-24

**Authors:** Dana Sonia Nagy, Alexandru Isaic, Alexandru Catalin Motofelea, Dorel Ionel Popovici, Razvan Gheorghe Diaconescu, Serban Mircea Negru

**Affiliations:** 1 Department of Oncology, Victor Babes University of Medicine and Pharmacy, Bd. Victor Babes No. 16, 300226 Timisoara, Romania; dana.nagy@oncohelp.ro (D.S.N.); dorel.popovici@umft.ro (D.I.P.); serban.negru@umft.ro (S.M.N.); 2 Department of Palliative Care, OncoHelp Hospital Timisoara, Ciprian Porumbescu Street, No. 59, 300239 Timisoara, Romania; 3 Department X of General Surgery, “Victor Babes” University of Medicine and Pharmacy, 300041 Timisoara, Romania; 4 Department of Internal Medicine, University of Medicine and Pharmacy “Victor Babeș”, 300041 Timișoara, Romania; alexandru.motofelea@umft.ro; 5 Department of Oncology, OncoHelp Hospital Timisoara, Ciprian Porumbescu Street, No. 59, 300239 Timisoara, Romania; razvan.diaconescu@umft.ro

**Keywords:** spirituality, religion, cancer patients, quality of life, coping, systematic review, yoga, mindfulness

## Abstract

Background/Objectives: This systematic review aimed to comprehensively evaluate the role of spirituality and religion in the journey of patients with cancer and assess their impact on various aspects of well-being and coping mechanisms. Methods: Systematic searches were conducted in PubMed, Scopus, and Google Scholar following the PRISMA guidelines. This study focused on the period from 2014 to 2024, the time chosen for the emerging integration of spirituality and religion in cancer treatment. Inclusion criteria targeted studies exploring the impact of spirituality and religion on cancer patients’ quality of life, coping, and treatment outcomes. Results: A comprehensive search initially yielded 2591 papers, of which 1544 were excluded as duplicates, and 113 were further excluded based on the inclusion criteria. Ultimately, 53 papers were selected for review, including 8 prospective cohort, 17 cross-sectional, 16 observational descriptive, and 12 RCT studies. Encompassing 13,590 patients with various cancer types, including breast, gastrointestinal, prostate, brain, and others, the review highlighted spirituality and religion’s significant role in improving cancer patients’ well-being. Across different cancers, greater spiritual well-being and religious coping were consistently associated with an improved quality of life, reduced distress, enhanced coping, and better treatment outcomes. Interventions such as mindfulness therapy, yoga, and religious coping strategies positively impact patients’ spiritual and emotional well-being. Conclusions: This review highlights the vital role of spirituality and religion in cancer care. Integrating these aspects into patient plans offers comfort and support throughout treatment. Healthcare providers should prioritize spiritual support to enhance patient well-being and optimize outcomes.

## 1. Introduction

Cancer remains a significant global health concern, with millions of new cases and deaths reported annually. In 2022, an estimated 20 million new cases of cancer and 9.7 million deaths were reported worldwide [1]. Despite advancements in treatment and prevention strategies, the burden of cancer continues to impact individuals and communities worldwide. However, it is noteworthy that approximately 53.5 million individuals survive within a five-year period after diagnosis, emphasizing the importance of effective management and support for cancer patients [1].

Efforts to combat cancer focus not only on prevention and treatment but also on improving the overall well-being and quality of life of patients. Prevention strategies targeting known risk factors can potentially avert 30 to 50% of cancer cases, highlighting the significance of public health interventions and lifestyle modifications. Moreover, early detection and timely treatment significantly enhance the chances of successful outcomes across various cancer types [2,3]. Currently, research has started focusing on less invasive approaches for the early diagnosis and the targeting of potential specific biomarkers for various types of malignancies [4]. Nevertheless, the journey of cancer diagnosis and treatment often imposes profound physical and emotional challenges for patients. Coping with the uncertainties, fears, and changes associated with cancer can significantly impact the well-being and quality of life of individuals affected by the disease [5]. In this context, spirituality and religion have emerged as potent coping mechanisms that offer solace, strength, and hope amidst the turmoil of illness.

Spirituality encompasses an individual’s quest for transcendence and meaning, engaging with existential questions regarding purpose, connection, and significance. It represents a deeply personal aspect of human experience, often intertwined with one’s values, beliefs, and relationships. Religion, on the other hand, refers to organized systems of beliefs and practices shared by communities, offering a framework for spiritual expression and communal support [6], whereas religiousness refers to the degree to which an individual believes and practices a religion and follows its dogma, often shaping how one choses to live and interact with the others [7]. Studies show that religious coping was a stronger predictor of outcomes during negative life events than general religiosity [8]. It is pertinent to recognize the significance of spirituality in all patients, irrespective of their religious affiliations, as it serves as a fundamental aspect of holistic care.

Moreover, practices such as yoga, which integrate physical postures, breathing exercises, and meditation, are increasingly recognized for their potential to enhance spirituality and well-being among cancer patients [9]. By fostering a connection between the body, mind, and spirit, yoga offers a holistic approach to coping with the challenges of cancer diagnosis and treatment.

While much of the literature focuses on the positive impact of spirituality and religion on health outcomes, it is important to emphasize that it can also have negative effects, particularly through negative religious coping. Negative religious coping involves maladaptive religious behaviors, such as spiritual struggle, anger with God, or feelings of abandonment, which have been associated with poorer quality of life (QoL) and increased psychological distress in some individuals. This aspect of spirituality and health highlights the complexity of the relationship between religious beliefs and well-being. Although this review focuses specifically on the positive outcomes of spiritual and religious support in cancer care, it is essential to acknowledge the existence of negative religious coping, as it underscores the need for an understanding of spirituality in health contexts [10,11,12].

This systematic review aims to explore the impact of spirituality and religion on the lives of cancer patients, emphasizing their role in coping strategies, quality of life, and mental health. By synthesizing existing evidence, we seek to demonstrate the importance of integration of spiritual and religious support into cancer care protocols. Recognizing the importance of patient-centered approaches, improved healthcare provider-patient communication, and holistic support can potentially enhance the efficacy of conventional cancer treatments and foster a more compassionate and comprehensive approach to cancer care.

## 2. Materials and Methods

To conduct a comprehensive systematic review on the role of spirituality and religion in providing reassurance to cancer patients, a rigorous methodology was employed.

### 2.1. Study Design and Search Strategy

A systematic search of electronic databases was conducted across PubMed, Scopus, and Google Scholar. This systematic review was registered in PROSPERO with the registration number CRD42024530357. Our investigation was centered around a 10-year time frame, from 2014 to 2024. This time frame was carefully chosen due to the relatively novel integration of spirituality and religion in the treatment of cancer patients, which presents a fresh approach for medical and clinical professionals. We strategically used the following search phrases including (“influence of spirituality AND cancer patients”), (“influence of religion AND cancer patients”), (“spirituality AND quality of life cancer”), (“religion AND quality of life cancer”), (“influence of spirituality AND coping cancer”), and (“influence of religion AND coping cancer”).

### 2.2. Inclusion/Exclusion Criteria

The inclusion criteria comprised studies with full-text availability, published in English, involving adult oncology patients across diverse cancer types and stages, all of whom were actively undergoing treatment, with reported positive outcomes. Exclusion criteria included studies focusing on populations other than cancer patients, such as caregivers or pediatric patients, as well as medical staff (nurses/doctors) and cancer patients in recovery. Review/meta-analysis studies, outcomes unrelated to spirituality/religion in cancer care, or studies not involving cancer patients were also excluded.

### 2.3. Data Extraction

Two independent reviewers assessed titles and abstracts for eligibility according to Preferred Reporting Items for Systematic Reviews and Meta-Analyses (PRISMA) guidelines. Eligible studies underwent full-text screening, and relevant data were extracted. Data included authorship, publication year (2014–2024), population characteristics (including cancer type and gender), sample size, measures of spirituality and religion, and outcomes related to reassurance, coping, quality of life, and mental health. In addition to commonly studied interventions like yoga and mindfulness, the review also considered other forms of spiritual practices such as prayer, nature-based experiences, and social connections, providing a comprehensive view of diverse strategies that promote spiritual well-being.

### 2.4. Data Synthesis and Analysis

The extracted data were synthesized and analyzed to identify common themes, patterns, and associations between spirituality, religion, and the experiences of cancer patients. Findings were organized systematically to provide a comprehensive overview of the role of spirituality and religion in offering reassurance throughout the cancer journey, including diagnosis, treatment, and survivorship. This synthesis aimed to elucidate the impact of spirituality and religion on coping strategies, quality of life, and mental well-being, contributing to a deeper understanding of their significance in cancer care.

### 2.5. Risk of Bias Assessment and Ethical Considerations

Methodological quality assessment of included studies was performed using appropriate tools, such as the Cochrane risk of bias tool for RCTs and the Newcastle–Ottawa Scale (NOS) for observational studies. The Cochrane risk of bias tool evaluates various aspects of trial design, execution, and documentation to assess the potential for bias in randomized controlled trials (RCTs). The NOS assesses the quality of non-randomized studies based on selection, comparability, and exposure or outcome determination.

Ethical approval was not required for this systematic review, as it involved the analysis of published literature and did not entail direct contact with human participants. All data were obtained from publicly available sources and were anonymized during analysis to ensure confidentiality and compliance with ethical standards.

## 3. Results

Literature search results

**Figure 1:**
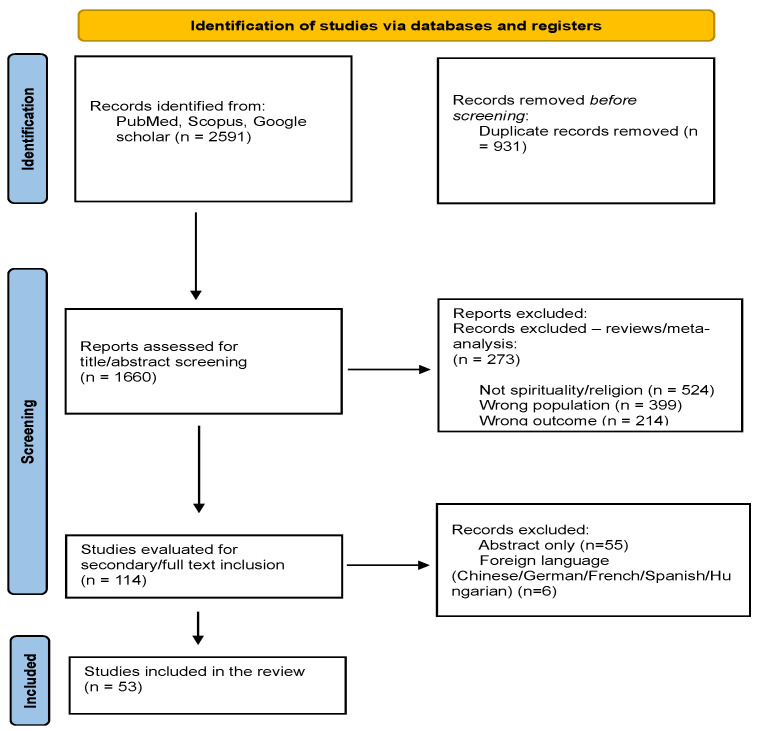
A PRISMA flowchart was created to summarize the selection process of the eligible studies. Potential content was extracted from selected studies.

Through a rigorous database search, a total of 2591 papers were initially identified. After removing 931 duplicates, 1544 studies were excluded as they did not meet the inclusion criteria upon review (Figure 1).

During the primary screening, exclusions were made based on criteria such as the absence of spirituality/religion (n = 524), incorrect population focus (n = 399), inappropriate outcome measures (n = 214), and studies not involving cancer patients (n = 136).

Following an assessment of titles and abstracts, 113 studies were selected for further evaluation in full-text format. Additionally, during the secondary screening, reports were excluded if they were available only as abstracts (n = 55) or were written in foreign languages including Chinese, German, French, Spanish, or Hungarian (n = 6). Consequently, only 53 papers were deemed suitable for inclusion in this review.

### 3.1. Study Characteristics

Of the 53 identified studies, 8 were prospective cohort, 17 were cross-sectional, 16 were observational descriptive, and 12 were randomized controlled trials (RCTs). The studies encompassed diverse populations and sample sizes across different years, reflecting a broad perspective on the impact of spirituality and religion on cancer patients’ well-being. Commonly reported positive outcomes included enhanced quality of life, emotional well-being, improved coping mechanisms, and reduced psychological distress. The most frequently used assessment tools for evaluating spirituality included the FACIT-Sp (Functional Assessment of Chronic Illness Therapy—Spiritual Well-Being), the Daily Spiritual Experience Scale (DSES), and the Spiritual Well-Being Scale (SWBS). Psychological outcomes were typically measured using tools such as the Hospital Anxiety and Depression Scale (HADS) and the Depression Anxiety Stress Scales (DASS-21), providing a comprehensive evaluation of both spiritual and mental health. Barata A. et al. [1] conducted research in 2022 involving 242 women with breast cancer undergoing chemotherapy. Majda A. et al. [2] surveyed 101 Polish cancer patients in 2021, with 101 correctly completed questionnaires out of 150 individuals approached. Zare A. et al. [5] investigated 208 Iranian and Muslim cancer patients receiving chemotherapy in 2019.

Zargani A. et al. [6] studied 84 Muslim patients with breast cancer in 2017. Zok A. et al. [9] focused on 41 breast cancer patients in 2023. Joshi A. et al. [13] included 32 breast cancer patients undergoing chemotherapy in their 2021 study. Ferrell B. et al. [14] conducted research with 479 cancer patients with solid tumors in 2020. The sample sizes and characteristics varied across studies, reflecting diverse populations and cancer types, providing a broad perspective on the impact of spirituality and religion on cancer patients’ well-being.

### 3.2. Summary of the Selected Studies

#### Breast Cancer Patients

According to the PRISMA methodology, 53 studies were included in the review (Table 1). Breast cancer patients were the most frequently studied population, with 14 studies focusing on this group. Breast cancer remains a significant health concern globally, and many patients face difficulties in coping with the disease and its treatment [13]. Interventions such as spirituality, mindfulness therapy, religiosity, and yoga were assessed for their potential benefits and broader application. These studies, collectively involving 929 participants, consistently demonstrated a positive correlation between greater spiritual well-being and improved quality of life, emotional well-being, enhanced functional capacity, and better social interactions [14,15,16,17]. Spirituality was seen as a critical source of hope and meaning for patients, helping them navigate the cancer journey. One study reported that low spiritual fulfillment was linked to higher levels of demoralization, underscoring the importance of addressing spiritual needs [18]. Group therapy was found to facilitate spiritual fulfillment among breast cancer patients, leading to enhanced quality of life [19].

Mindfulness therapy was evaluated in two studies, resulting in significantly reduced psychological distress and improved spiritual well-being, thus enhancing overall quality of life [20,21]. On the other hand, religiosity emerged as a significant coping mechanism, with three studies investigating its impact on quality of life among breast cancer patients of various faiths, demonstrating higher levels of well-being among those with greater religiosity [22,23,24]. Similarly, spirituality and mindfulness practices, particularly through yoga, were associated with pain reduction, increased quality of life, reduced psychological distress, and improved coping and sense of purpose in life [25,26,27].

### 3.3. Other Cancer Types

Three studies focusing on patients with gynecological cancer were included in our review, comprising a total of 932 participants. These studies found that both spirituality and religion, while often interconnected, positively influenced various aspects such as illness perception, quality of life, existentiality, and one’s relationship with something greater than themselves [28,29,30]. This suggests that spirituality and religious beliefs play a significant role in coping with the challenges of gynecological cancer, providing patients with a sense of meaning and support during their journey.

In the case of gastrointestinal cancer, two studies were identified. One of these studies utilized fatwas, which are Islamic legal opinions or rulings issued by qualified scholars in response to specific questions about Islamic law. The study aimed to enhance participants’ spiritual well-being and quality of life through this culturally tailored intervention. This approach was viewed as an important coping mechanism, potentially reducing the stigma associated with treatment and disease [31]. The second study explored the role of spirituality in improving life quality and overall well-being among patients with gastrointestinal cancer, showing positive outcomes. These findings highlight the potential benefits of incorporating spirituality into coping strategies for gastrointestinal cancer patients [32].

Brain cancer was the subject of two studies included in the review, involving a total of 633 participants. The results indicated that greater spiritual well-being and religious coping were associated with better health-related quality of life for patients in this category. This suggests that spirituality may serve as a valuable resource for individuals facing the challenges of brain cancer, offering comfort and support throughout their journey [33,34].

Two studies focused on patients with solid tumors, comprising a total of 679 cases. The findings suggested that higher levels of spirituality were associated with higher quality of life and support, as well as reduced severity of fatigue and overall symptom interference. This underscores the importance of addressing the spiritual needs of patients with solid tumors to enhance their well-being and coping mechanisms [35,36].

Additionally, one study investigated the impact of yoga practice on spiritual well-being and quality of life among patients with prostate cancer. The results revealed a positive correlation between yoga practice and emotional, physical, and social scores, indicating the potential benefits of integrating yoga into holistic cancer care approaches [37].

Lastly, a cohort of 864 patients with lung cancer was evaluated, demonstrating that increased spirituality was associated with decreased emotional distress. This suggests that spirituality may serve as a protective factor against emotional distress among patients with lung cancer, highlighting the importance of addressing spiritual needs in comprehensive cancer care plans [38]. The studies reviewed demonstrate that both spirituality and religion enhance the quality of life of cancer patients. Spirituality contributes to a sense of meaning and purpose, and religion frequently offers structured coping mechanisms, both playing significant roles in the well-being of the cancer patients.

### 3.4. General Cancer Population

For the remaining 28 studies included in the review, the patient cohort did not specify the cancer type. However, 11 studies (involving 4183 participants) investigated the influence of spirituality on participants’ well-being and quality of life. These studies reported positive outcomes, including stronger social support, enhanced quality of life, reduced neuropathic pain, decreased psychological distress, fatigue, and depression, as well as improved overall spiritual health [39,40,41,42,43,44,45,46,47,48,49]. Another 11 studies focused on the impact of religiosity and prayer, highlighting their effects on various aspects of cancer patients’ lives. With a total population of 4495, these studies demonstrated improvements in pain management, coping mechanisms, social support, quality of life, optimism, and mental health [50,51,52,53,54,55,56,57,58,59,60].

Additionally, four studies examined the effects of spirituality through yoga practice on 337 patients, showing positive outcomes such as reduced fatigue, physical and psychosocial distress, anxiety, and increased quality of life [36,46,48,50,53]. Furthermore, mindfulness therapy was evaluated in two studies involving 165 participants, resulting in reduced suffering scores and improved life quality [61,62].

### 3.5. Assessment Tools Identified in the Studies

Spirituality and religion were assessed using different tools (Table 2). The studies revealed diverse impacts of spirituality and religion on cancer patients’ well-being. Barata A. et al. [14] associated higher spiritual well-being (FACIT-Sp) with decreased distress and enhanced quality of life in women undergoing chemotherapy.

Majda A. et al. [2] found a positive link between spiritual experiences (DSES) and well-being among Polish cancer patients. Zare A. et al. [5] linked elevated spiritual well-being, mental health, and quality of life (DASS-21) with improved overall well-being in Iranian and Muslim cancer patients. Zok A. et al. [9] noted increased satisfaction post-yoga, targeting spiritual well-being. Joshi A. et al. [13] showcased MBAT’s efficacy in reducing distress and enhancing spiritual well-being in breast cancer patients. Ferrell B. et al. [14] and Randazzo D. et al. [17] highlighted spirituality’s importance in patients’ quality of life (FACIT-Sp12). Similarly, Dabo I. et al. [24] linked Croatian cancer patients’ well-being (EORTC QLQ-SWB32) with spiritual well-being, emphasizing spirituality’s multifaceted role in coping.

Although not included in the studies mentioned before, the Spiritual Needs Questionnaire (SpNQ) is a widely used tool designed to assess the spiritual needs of patients, particularly in palliative and cancer care. It explores various dimensions of spirituality, including existential issues, hope, and the need for spiritual support, offering a comprehensive approach that goes beyond religious practices. The SpNQ is validated across multiple cultural contexts, demonstrating its adaptability and relevance in diverse healthcare settings. One of its strengths is its holistic view of spirituality, recognizing both religious and non-religious aspects, which allows it to capture the full range of spiritual concerns patients may face. This makes it especially valuable in cancer care, where patients often seek meaning and emotional support. Additionally, the tool has been widely applied in countries such as the Netherlands, Japan, and the UK, further supporting its global applicability. However, the SpNQ, like any self-report instrument, may be influenced by personal biases and cultural factors, which could impact its accuracy in certain patient groups. Despite these limitations, the SpNQ remains a useful and reliable instrument for identifying spiritual needs and informing spiritual care interventions in cancer treatment [63,64].

### 3.6. Risk of Bias Assessment

After conducting a comprehensive analysis of the studies included in the systematic review, it was determined that the risk of bias ranged from low to medium across all types of studies considered. The NOS was utilized for cohort and cross-sectional studies, while the Cochrane risk of bias tool was employed for RCTs. This approach ensured a rigorous evaluation of study quality and minimized the potential for bias in the findings. By utilizing established assessment tools tailored to the study designs, the review was able to establish a robust and reliable foundation for the conclusions drawn from the data. As a result, the credibility of the results obtained is strengthened, instilling confidence in the reliability of the findings presented in the systematic review.

## 4. Discussion

### 4.1. Summary of Evidence

This study conducted a comprehensive systematic review, encompassing 53 diverse studies that collectively evaluated 13,590 patients diagnosed with various types of cancer. The primary objective of these investigations was to delve into the impact of spirituality and religion on the overall well-being and quality of life of individuals undergoing cancer diagnosis and treatment. Despite the advancements in technology and treatment modalities within contemporary medical practice, the significance of spiritual and compassionate care remains paramount. This holistic approach not only addresses the physical aspects of illness but also attends to the emotional, social, and spiritual needs of patients, recognizing the profound influence of spirituality on coping with challenging diagnoses [65].

A total of 25 included studies focused on exploring the role of spiritual well-being as a coping mechanism for individuals grappling with cancer. These studies consistently demonstrated that heightened spirituality correlates with improved quality of life, enhanced coping mechanisms, and reduced psychological distress. The profound influence of spirituality on psychological well-being underscores its significance in shaping health-related behaviors and perceptions, bridging the realms of physical health and psychosocial well-being [66]. In various studies, the spiritual care needs of patients were addressed by a range of professionals, including healthcare staff, spiritual care specialists, and even volunteers. This highlights that patients’ spiritual needs can be met through support from diverse individuals with different perspectives, all having specific roles in achieving the patient’s well-being.

Among the interventions explored, yoga practice emerged as particularly promising, offering benefits not only for physical and mental well-being but also for spiritual well-being. Eight studies within the review highlighted the effectiveness of yoga in enhancing satisfaction levels, fostering self-compassion, and mitigating psychological distress, anxiety, and neuropathic pain. The holistic approach of yoga nurtures both body and mind, cultivating a state of equanimity in perception that complements conventional interventions like analgesia in managing chronic pain associated with cancer [67,68].

The interconnection of spirituality and mindfulness was a notable theme across the included studies. While spirituality embodies a state of mindfulness, mindfulness entails the deliberate cultivation of spirituality. Both concepts are deeply intertwined. Four studies within the review underscored the beneficial effects of mindfulness, showcasing its potential to reduce overall suffering scores and enhance quality of life for cancer patients [69].

Religion emerged as a consistent and significant coping mechanism for individuals navigating life-threatening illnesses such as cancer. The review included 15 studies examining the impact of religiosity and faith on the lives of cancer patients. These studies collectively revealed a positive influence of religion on various aspects of well-being, including improved quality of life, enhanced coping mechanisms, and bolstered physical and mental health outcomes [70].

Although most studies emphasize the beneficial effect religion and spirituality have on the QoL of cancer patients, several studies have raised important negative aspects regarding the measurement of spirituality and well-being in healthcare research. Koenig (2008) [71] highlighted that the definition of spirituality has broadened, often incorporating traits like optimism, forgiveness, and well-being, leading to measures that overlap with mental health. He argued that such overlap results in tautological findings and called for either a clearer, traditional definition or the elimination of spirituality as a research construct.

Similarly, Visser et al. (2017) [72] found that existential well-being (EWB), commonly included in spirituality measures, overlaps with psychological well-being. Using the Spiritual Attitude and Involvement List (SAIL), they showed that traits like coping belong to well-being, while meaning in life aligns more with spirituality. They advocate for distinct measures to better assess the relationship between these constructs.

Future research would benefit from the development of more nuanced and culturally sensitive tools that can better assess the role of spirituality in health outcomes.

Another important aspect that needs to be addressed is the role of spirituality and religion in palliative care. Palliative care plays a critical role in supporting cancer patients, particularly those facing advanced or terminal stages of their illness. As defined by the World Health Organization (WHO), palliative care focuses on improving the quality of life by addressing the physical, psychological, emotional, and spiritual needs of patients. In the context of cancer, patients often experience profound existential distress, fear of death, and emotional suffering, which can severely affect their well-being [73]. Spiritual care, an essential part of palliative care, provides patients with a means of finding meaning, hope, and peace amidst their illness. Interventions such as religious support, spiritual counseling, and mindfulness practices are specifically designed to help patients navigate these emotional and existential challenges. Despite its significance, spiritual care remains underexplored in research on cancer patients within palliative care settings [74]. Studies in the field of cancer care focus primarily on the medical and physical aspects of treatment, neglecting the emotional and spiritual needs of patients [74,75,76]. This gap in research highlights the need for a more holistic approach to palliative care, one that fully integrates spiritual care as a core component. Spiritual care is not a peripheral aspect of care but is central to improving the quality of life for cancer patients, particularly in the final stages of their illness [76]. It is crucial to further investigate how spiritual care can be effectively integrated into palliative care to enhance the well-being of cancer patients, alleviate psychological distress, and improve their overall quality of life, particularly in their final stages of life. More research is needed to ensure that palliative care fully meets the spiritual and emotional needs of cancer patients, providing holistic and compassionate care that goes beyond physical symptom management.

In summary, the findings from this systematic review underscore the profound influence of spirituality and religion on the well-being and quality of life of individuals grappling with cancer. From heightened spiritual well-being to the therapeutic benefits of yoga and mindfulness practices, as well as the resilience offered by religiosity, these insights provide valuable avenues for holistic care and support for cancer patients.

### 4.2. Limitations of This Review

The primary limitation of this review is the restricted access to sources, as many full-text unavailable studies were excluded, potentially limiting the scope and conclusions. Additionally, focusing solely on patients after diagnosis or during treatment may have limited diversity in perspectives. While this approach enhanced result accuracy by considering coping mechanisms’ psychological implications, it excluded perspectives from recovered patients. Future research could benefit from expanding inclusion criteria to encompass recovered patients and accessing a broader range of sources. Expanding this approach could yield a more comprehensive understanding, providing valuable insights to inform oncology clinical practices and decisions.

### 4.3. Practical Implications and Future Directions

The primary objective of this review was to emphasize the significance of integrating spirituality/religion into the care of individuals diagnosed with cancer, in accordance with their personal preferences and beliefs. Integrating spirituality or religion into cancer care can provide patients with solace, optimism, and a sense of purpose during challenging, emotionally taxing times. Research indicates that attending to the spiritual needs of patients can enhance their quality of life, overall well-being, and treatment outcomes. By incorporating these dimensions into patient care plans, healthcare providers can adopt a more comprehensive approach that acknowledges the interconnectedness of the mind, body, and spirit. It is imperative to honor each individual’s beliefs and choices to provide comprehensive support throughout their cancer journey.

## 5. Conclusions

This review highlights spirituality and religion’s vital role in cancer patients’ lives. Engaging in these practices provides profound reassurance, enhancing well-being and quality of life while alleviating symptoms like pain, depression, and anxiety. Integrating spirituality and religion into care plans offers a holistic approach, addressing patients’ multifaceted needs. These practices not only enhance the overall well-being and quality of life of patients but also serve as powerful coping mechanisms, alleviating symptoms such as neuropathic pain, depression, and anxiety. By acknowledging and integrating spirituality and religion into patient care plans, healthcare providers can adopt a more holistic approach that addresses the multifaceted needs of cancer patients. Healthcare providers must recognize its significance in cancer care, fostering resilience and hope throughout the treatment journey. This review emphasizes the need to incorporate spirituality into the broader framework of cancer care, ensuring that patients receive comprehensive support during their challenging battle against cancer.

## Figures and Tables

**Table 1:** Overview of the studies assessing spirituality/religion in cancer patients.

Author	Year	Population	Population Size	Strategy Assessment (Spirituality/Religion)	Outcome	Cultural Backgrounds
Barata A. et al. [1]	2022	Women with breast cancer undergoing chemotherapy	242	Spiritual well-being (using FACIT-Sp) Psychological distress (using HADS) Quality of life (using FACT-G)	Greater spiritual well-being is associated with less concurrent distress and better quality of life, as well as with greater emotional, functional, and social well-being over time	Hispanic culture
Majda A. et al. [2]	2021	Polish cancer patients, both men and women	Enrolled = 150, Completed = 101	Daily Spiritual Experience Scale (DSES)	The level of spirituality/religiosity was positively and significantly correlated with quality of life (*p* < 0.001), physical functioning (*p* < 0.048), fulfilling one’s role (*p* < 0.004), emotional functioning (*p* < 0.001) and social functioning (*p* < 0.001)	Central-Eastern Europe, Polish culture
Zare A. et al. [5]	2019	Iranian and Muslim cancer patients receiving chemotherapy	208	Spiritual well-being, mental health, and quality of life using DASS-21 questionnaire	Participants with higher level of spiritual well-being indicated higher level of mental health (*p* = 0.001) and quality of life (*p* = 0.01)	Muslim culture
Zargani A. et al. [6]	2017	Muslim patients with breast cancer	84	Serajzadeh’s Muslim Religiosity questionnaire	Quality of life was significantly higher in patients with high religiosity status compared to moderate religiosity status (*p* < 0.0001); and a direct correlation between QoL and religiosity (total and all subscales) (*p* < 0.0001)	Muslim culture
Zok A. et al. [9]	2023	Breast cancer patients	41	Spiritual well-being using 60-min vinyasa yoga practice followed by 15-min shavasana relaxation	After three months of exercise, the participants’ satisfaction levels improved significantly	Central-Eastern Europe, Polish culture
Joshi A. et al. [13]	2021	Breast cancer patients undergoing chemotherapy	32	Mindfulness-Based Art Therapy (MBAT) for improving spiritual well-being	MBAT intervention for breast cancer patients undergoing chemotherapy significantly decreased the psychological distress, improved the spiritual well-being in terms of meaning, peace, faith.	Indian culture
Ferrell B. et al. [14]	2020	Cancer patients (with solid tumors)	479	FACIT-Sp12 assessment tool	Spirituality is a key aspect of the quality of life and a source of support during serious illness and treatment	Australian culture
Kimani C. et al. [16]	2023	Cancer patients	169	Determinants of progressive utilization of palliative care services by cancer patients	Muslim religion was a significant factor in palliative care utilization	Kenyan culture
Kaushik D. et al. [15]	2021	Prostate cancer patients	29	Spiritual well-being and quality of life using yoga practice	Yoga has a positive effect on emotional, physical, and social scores	American culture (USA)
Randazzo D. et al. [17]	2021	Brain tumor patients	606	FACIT-Sp12 assessment tool	Greater spiritual well-being was associated with better health-related quality of life (*p* < 0.0001)	American culture (USA)
Vilhena E. et al. [18]	2014	Patients with chronic disease, including cancer	774	Self-reporting questionnaires to assess general well-being, physical and mental health	For the cancer disease group, positive effect and spirituality showed a statistically significant association with general well-being, mental health, and subjective well-being (*p* < 0.01)	Portuguese culture
Ahmadi F. et al. [19]	2019	Cancer patients in Malaysia	29	Religious coping methods assessed	Malay culture and Islamic belief strongly influences coping methods of cancer patients	Turkish culture
Iqbal F. et al. [20]	2016	Patients with colorectal cancer and stoma	10	Religious coping using stoma-specific non-binding legal opinions (fatawas)	These fatawas are a way to improve quality of life in patients with stomas	Muslim culture
Cheng F. [21]	2017	Case study	1	Religious impact on pain reduction	Buddhism underpins pain tolerance and therefore reduces pain intensity	Buddhist culture
Rong H. et al. [22]	2022	Cancer patients	208	Assessing the relationship between social support and benefit finding as mediated by perceptions of spirituality	Spirituality mediated the effect of social support on benefit finding (*p* < 0.01)	Chinese culture
Hekmati P. et al. [23]	2015	Cancer patients undergoing chemotherapy	96	Relationship between praying and life expectancy	There was a significant relationship between praying and life expectancy (*p* = 0.01)	Iranian culture
Dabo I. et al. [24]	2021	Croatian cancer patients	143	Assessing spiritual well-being using EORTC QLQ-SWB32	Women and older patients scored higher on RG scale (Relationship to God) and spiritual well-being	Croatian culture
Bai J. et al. [25]	2018	Black patients with cancer pain	102	Relationship between spirituality, quality of life and pain	Higher spirituality was associated with lower pain severity (*p* = 0.01) and significantly predicted quality of life domains of social (*p* < 0.0001), emotional (*p* = 0.002), and functional well-being (*p* = 0.001)	American culture (USA)
Chen J. et al. [26]	2021	Patients with gynecological cancer in China	705	Spiritual well-being in relation to quality of life	Being religious was associated with higher scores on the existential (EX) and relationship with someone greater (RSG) scale	Chinese culture
Heidari J. et al. [27]	2015	Digestive system metastatic cancer	250	Spiritual well-being in relation to quality of life	Significant relationship between spirituality and life quality scape (*p* < 0.001)	Iranian culture
Carson J. et al. [28]	2021	Women with metastatic breast cancer	48	Mindful yoga program to reduce pain	Meditation practice showed the strongest association with lower daily pain	American culture (USA)
Piderman K. et al. [29]	2015	Patients with brain cancer	27	Chaplain-led spiritual life interview to assess spiritual well-being, spiritual coping and quality of life in cancer patients	Increases in spiritual well-being, religious coping, and quality of life were detected	American culture (USA)
Nasution L. et al. [30]	2019	Patients with gynecological cancer	108	Measuring spiritual well-being in cancer patients	Positive change in the average scores of coping (*p* = 0.001) and spiritual well-being after receiving spiritual intervention (*p* = 0.006)	Indonesian culture
Gudenkauf L. et al. [31]	2019	Lung cancer patients	864	Relationship among spirituality, emotional distress of cancer patients	Spirituality was associated with lower prevalence of emotional distress	American culture (USA)
Amzerin M. et al. [32]	2020	Moroccan cancer patients	164	Assessing quality of life in cancer patients	Patients with abnormal G8 scores (geriatric assessment with 8 questions) did less standing prayer and had lower scores of physical, emotional and social functions	Moroccan culture
Jetan M. et al. [33]	2023	Cancer patients	354	FACIT-Sp12	A strong positive correlation between spirituality and quality of life was observed	Palestinian culture
Miller M. et al. [34]	2021	Solid tumor cancer patients	200	Spirituality and cancer-related symptoms	Higher spirituality was associated with lower severity of fatigue (*p* = 0.003) and lower overall symptom interference (*p* < 0.004)	American culture (USA)
Wnuk M. [35]	2022	Breast or lung cancer patients	50	Daily Spiritual Experience Scale (DSES) Purpose in Life Scale (PIL) Herth Hope Index (HHI) and CantrilLadded	Spiritual experiences were indirectly related to life satisfaction through the pathway of hope and meaning in life	Central-Eastern European Polish Culture
McLaughlin E. et al. [36]	2024	Young adults with cancer	28	8-week yoga intervention	Participants described improvements in their ability to practice self-care, self-compassion and mindfulness both during and beyond yoga classes	Canadian culture
Delgado-Guay M. et al. [37]	2021	Latin American cancer patients	325	Faith, Importance and Influence, Community, and Address (FICA)	Spirituality/religiosity was associated with positive brief coping strategies and higher quality of life (*p* < 0.0001)	Latin American culture
Calderon C. et al. [38]	2020	Cancer patients	912	FACIT-Sp	Meaning/peace and faith are correlated positively with optimism and social support	Hispanic culture (Spain)
Hoseini L. et al. [39]	2016	Breast cancer patients	176	Spiritual well-being scale (SWBS)	Spiritual well-being and social support had direct effects on quality of life and illness perception	Iranian culture
Ahmadi N. et al. [40]	2017	Cancer patients	2355	Survey study on the role of religion in coping	The people in the oldest age-group, women and people raised in places with 20,000 or fewer residents showed a higher average use of religious coping mechanisms thank younger people, men and those raised in larger towns	Swedish culture
Kugbey N. et al. [41]	2020	Breast cancer patients from Ghana	11	Spirituality coping	Reliance on spirituality, religious faith and social support are the main coping strategies	Ghana culture
Prince P. et al. [42]	2015	Hispanic allogeneic hematopoietic stem cell transplant patients	171	Spirituality well-being (FACIT-SpWB)	Hispanic patients enjoyed greater spiritual well-being compared to non-Hispanic counterparts	Hispanic culture
Phenwan T. et al. [43]	2019	Breast cancer patients	16	Spiritual well-being (SWB)	Religion, especially Buddhism, is part of a larger holism of their spiritual well-being	Buddhist culture
Mohebbifar R. et al. [44]	2015	Patients with cancer	210	Spiritual health and quality of life assessment	Positive and significant relationship between spiritual health and quality of life in patients with cancer	Iranian culture
Moyses R. et al. [45]	2023	Amazonian women with cervical cancer under treatment	119	The role of spirituality in the relationship between psychological morbidity and quality of life	Spirituality moderated the relationship between illness perception and quality of life and between psychological morbidity and quality of life	Latin American culture
Mallaiah S. et al. [46]	2022	Cancer patients	95	Yoga as a mind-body practice for improving physical and mental health	A single yoga therapy intervention contributed to a significant improvement in global, physical, and psychosocial distress	American culture (USA)
Park S. et al. [47]	2017	Breast cancer patients	12	Mindfulness based cognitive therapy (MBCT)	MBCT had a favorable effect on the psychological status and quality of life	Japanese culture
Tan S. et al. [48]	2022	Cancer patients	73	Mindfulness based supportive therapy (MBST)	Statistically significant reduction in suffering score in the MBST group	Malaysian culture
Oliveira S. et al. [49]	2021	Cancer patients	49	Spiritual well-being scale (SWBS) Spirituality, religiousness and personal beliefs (SRPB)	Patients with a higher level of spirituality has less intense neuropathic pain (*p* < 0.05)	Brazilian culture
Zetzl T. et al. [50]	2020	Patients with different types of cancer	173	8-week yoga therapy	Yoga therapy resulted in a stronger reduction of general fatigue (*p* = 0.03), depression (*p* < 0.001), and stronger increase in quality of life (*p* = 0.002)	German culture
Chang TG. et al. [51]	2022	Breast cancer patients	121	Demoralization in relation to spiritual interest and quality of life	High demoralization was associated with low spiritual fulfillment and reduced quality of life	Taiwan culture
Tsai TJ. et al. [52]	2016	Cancer patients	200	Effect of religion on health	Religious participation stimulated positive emotions in cancer patients and contributed to the spiritual and mental comfort	Taiwan culture
Tan TT. et al. [53]	2021	Cancer patients	92	Mindful gratitude journaling on the quality of life	1 week of mindful journaling reduces the overall suffering score (*p* = 0.0001)	Malaysian culture
Velasco-Durantez V. et al. [54]	2024	Cancer patients	1807	Assessment of psychological distress, coping and spirituality of cancer patients	Coping and spirituality reduce psychological distress in cancer patients	Hispanic culture (Spain)
Liu W. et al. [55]	2022	Breast cancer patients	136	Mindfulness yoga and health-related quality of lfie	Mindfulness yoga is effective in improving mental health and quality of life	Chinese culture
Zhi WI. et al. [56]	2021	Cancer patients	41	Yoga on health-related quality of life	Yoga therapy was associated with reduced anxiety and reduced chemotherapy-induced peripheral neuropathic pain	American culture (USA)
Shi X. et al. [57]	2023	Cancer patients	200	Spiritual needs of cancer patients	Spiritual needs were significantly correlated with cancer-related fatigue, depression, and social support (*p* < 0.007)	Chinese culture
Sun XH. et al. [58]	2021	Cancer patients	100	Spiritual care and quality of life	Provision of spiritual care to patients with advanced cancer significantly improves their overall spiritual health (*p* = 0.002)	Chinese culture
Guo YQ. et al. [59]	2022	Breast cancer patients	20	Coping mechanisms assessment	Hope and developing spiritual growth can improve women’s quality of life	Chinese culture
Zamaniyan S. et al. [60]	2016	Breast cancer patients	24	Spiritual group therapy and spiritual well-being	Spiritual group therapy (religious and existential health) improved the quality of life and spiritual well-being	Iranian culture

**Table 2:** Assessment tools identified in the studies.

FACIT-Sp	Functional Assessment of Chronic Illness Therapy—Spiritual Well-Being
HADS	Hospital Anxiety and Depression Scale
DSES	Daily Spiritual Experience Scale
FACT-G	Functional Assessment of Cancer Therapy—General
DASS	Depression Anxiety Stress Scales
EORTC QLQ-SWB32	European Organization for Research and Treatment of Cancer—Spiritual Well-Being Questionnaire
PIL	Purpose In Life
SWBS	Spiritual Well-Being Scale
FICA	F: Faith or Beliefs, I: Importance or influence, C: Community, A: Address
SRBP	Spirituality, religiousness, and personal beliefs

## References

[1] Barata A., Hoogland A.I., Small B.J., Acevedo K.I., Antoni M.H., Gonzalez B.D., Jacobsen P.B., Lechner S.C., Tyson D.M., Meade C.D. (2022). Spiritual Well-Being, Distress and Quality of Life in Hispanic Women Diagnosed with Cancer Undergoing Treatment with Chemotherapy. Psychooncology.

[2] Majda A., Szul N., Kołodziej K., Wojcieszek A., Pucko Z., Bakun K. (2022). Influence of Spirituality and Religiosity of Cancer Patients on Their Quality of Life. Int. J. Environ. Res. Public Health.

[3] Roi A., Roi C.I., Andreescu N.I., Riviş M., Badea I.D., Meszaros N., Rusu L.C., Iurciuc S. (2020). Oral Cancer Histopathological Subtypes in Association with Risk Factors: A 5-Year Retrospective Study. Rom. J. Morphol. Embryol..

[4] Roi A., Boia S., Rusu L.-C., Roi C.I., Boia E.R., Riviș M. (2023). Circulating miRNA as a Biomarker in Oral Cancer Liquid Biopsy. Biomedicines.

[5] Zare A., Bahia N.J., Eidy F., Adib N., Sedighe F. (2019). The Relationship Between Spiritual Well-Being, Mental Health, and Quality of Life in Cancer Patients Receiving Chemotherapy. J. Fam. Med. Prim. Care.

[6] Zargani A., Nasiri M., Hekmat K., Abbaspour Z., Vahabi S. (2018). A Survey on the Relationship between Religiosity and Quality of Life in Patients with Breast Cancer: A Study in Iranian Muslims. Asia Pac. J. Oncol. Nurs..

[7] Vitorino L.M., Lucchetti G., Leão F.C., Vallada H., Peres M.F.P. (2018). The Association Between Spirituality and Religiousness and Mental Health. Sci. Rep..

[8] Zwingmann C., Klein C., Büssing A. (2011). Measuring Religiosity/Spirituality: Theoretical Differentiations and Categorization of Instruments. Religions.

[9] Zok A., Matecka M., Zapala J., Izycki D., Baum E. (2023). The Effect of Vinyasa Yoga Practice on the Well-Being of Breast-Cancer Patients During COVID-19 Pandemic. Int. J. Environ. Res. Public Health.

[10] Chaar E.A., Hallit S., Hajj A., Aaraj R., Kattan J., Jabbour H., Khabbaz L.R. (2018). Evaluating the Impact of Spirituality on the Quality of Life, Anxiety, and Depression among Patients with Cancer: An Observational Transversal Study. Support. Care Cancer.

[11] Bovero A., Leombruni P., Miniotti M., Rocca G., Torta R. (2016). Spirituality, Quality of Life, Psychological Adjustment in Terminal Cancer Patients in Hospice. Eur. J. Cancer Care.

[12] Kandasamy A., Chaturvedi S.K., Desai G. (2011). Spirituality, Distress, Depression, Anxiety, and Quality of Life in Patients with Advanced Cancer. Indian J. Cancer.

[13] Joshi A.M., Mehta S.A., Pande N., Mehta A.O., Randhe K.S. (2021). Effect of Mindfulness-Based Art Therapy (MBAT) on Psychological Distress and Spiritual Wellbeing in Breast Cancer Patients Undergoing Chemotherapy. Indian J. Palliat. Care.

[14] Ferrell B., Chung V., Koczywas M., Borneman T., Irish T.L., Ruel N.H., Azad N.S., Cooper R.S., Smith T.J. (2020). Spirituality in Cancer Patients on Phase 1 Clinical Trials. Psychooncology.

[15] Kaushik D., Shah P.K., Mukherjee N., Ji N., Dursun F., Kumar A.P., Thompson I.M., Mansour A.M., Jha R., Yang X. (2022). Effects of Yoga in Men with Prostate Cancer on Quality of Life and Immune Response: A Pilot Randomized Controlled Trial. Prostate Cancer Prostatic Dis..

[16] Kimani C.W., Kioko U.M., Ndinda C., Adebayo P.W. (2023). Factors Influencing Progressive Utilization of Palliative Care Services among Cancer Patients in Kenya: The Case of Nairobi Hospice. Int. J. Environ. Res. Public Health.

[17] Randazzo D.M., McSherry F., Herndon J.E., Affronti M.L., Lipp E.S., Miller E.S., Woodring S., Healy P., Jackman J., Crouch B. (2021). Spiritual Well-Being and Its Association with Health-Related Quality of Life in Primary Brain Tumor Patients. Neurooncol. Pr..

[18] Vilhena E., Pais-Ribeiro J., Silva I., Pedro L., Meneses R.F., Cardoso H., da Silva A.M., Mendonça D. (2014). Psychosocial Factors as Predictors of Quality of Life in Chronic Portuguese Patients. Health Qual. Life Outcomes.

[19] Ahmadi F., Erbil P., Ahmadi N., Cetrez Ö.A. (2019). Religion, Culture and Meaning-Making Coping: A Study Among Cancer Patients in Turkey. J. Relig. Health.

[20] Iqbal F., Zaman S., Karandikar S., Hendrickse C., Bowley D.M. (2016). Engaging with Faith Councils to Develop Stoma-Specific Fatawās: A Novel Approach to the Healthcare Needs of Muslim Colorectal Patients. J. Relig. Health.

[21] Cheng F.K. (2017). Cancer-Induced Bone Pain Management Through Buddhist Beliefs. J. Relig. Health.

[22] Rong H., Yin M., Ren P., Li Y., Qu H., Chen X. (2023). Spirituality as a Mediator Between Social Support and Benefit Finding Among Advanced Cancer Patients. Cancer Nurs..

[23] Hekmati Pour N., Hojjati H. (2015). The Relationship between Praying and Life Expectancy in Cancerous Patients. J. Med. Life.

[24] Dabo I., Skočilić I., Vivat B., Belac-Lovasić I., Sorta-Bilajac Turina I. (2021). Spiritual Well-Being for Croatian Cancer Patients: Validation and Applicability of the Croatian Version of the EORTC QLQ-SWB32. Int. J. Environ. Res. Public Health.

[25] Bai J., Brubaker A., Meghani S.H., Bruner D.W., Yeager K.A. (2018). Spirituality and Quality of Life in Black Patients With Cancer Pain. J. Pain Symptom Manag..

[26] Chen J., You H., Liu Y., Kong Q., Lei A., Guo X. (2021). Association Between Spiritual Well-Being, Quality of Life, Anxiety and Depression in Patients with Gynaecological Cancer in China. Medicine.

[27] Heidari J., Jafari H., Janbabaei G. (2015). life quality related to spiritual health and factors affecting it in patients afflicted by digestive system metastatic cancer. Mater. Socio Medica.

[28] Carson J.W., Carson K.M., Olsen M., Sanders L., Westbrook K., Keefe F.J., Porter L.S. (2021). Yoga Practice Predicts Improvements in Day-to-Day Pain in Women with Metastatic Breast Cancer. J. Pain Symptom Manag..

[29] Piderman K.M., Breitkopf C.R., Jenkins S.M., Euerle T.T., Lovejoy L.A., Kwete G.M., Jatoi A. (2015). A Chaplain-Led Spiritual Life Review Pilot Study for Patients with Brain Cancers and Other Degenerative Neurologic Diseases. Rambam Maimonides Med. J..

[30] Nasution L.A., Afiyanti Y., Kurniawati W. (2020). Effectiveness of Spiritual Intervention Toward Coping and Spiritual Well-Being on Patients with Gynecological Cancer. Asia Pac. J. Oncol. Nurs..

[31] Gudenkauf L.M., Clark M.M., Novotny P.J., Piderman K.M., Ehlers S.L., Patten C.A., Nes L.S., Ruddy K.J., Sloan J.A., Yang P. (2019). Spirituality and Emotional Distress Among Lung Cancer Survivors. Clin. Lung Cancer.

[32] Amzerin M., Layachi M., Bazine A., Aassab R., Arifi S., Benbrahim Z., Khmamouche M.R., Kairouani M., Raiss H., Majid N. (2020). Cancer in Moroccan Elderly: The First Multicenter Transverse Study Exploring the Sociodemographic Characteristics, Clinical Profile and Quality of Life of Elderly Moroccan Cancer Patients. BMC Cancer.

[33] Jetan M., Daifallah A., Rabayaa M.K., Qadri R., Nassorah M., Nouri A., Al-Othaman N. (2023). The Impact of Spiritual Well-Being on the Quality of Life of Cancer Patients: A Cross-Sectional Study. Integr. Cancer Ther..

[34] Miller M., Kwekkeboom K., Cherwin C. (2022). The Role of Spirituality in Symptom Experiences Among Adults with Cancer. Support. Care Cancer.

[35] Wnuk M. (2022). Beneficial Effects of Spiritual Experiences and Existential Aspects of Life Satisfaction of Breast and Lung Cancer Patients in Poland: A Pilot Study. J. Relig. Health.

[36] McLaughlin E., Arshad N., Ellis K., Chen A., Fougere K., Culos-Reed S.N., Wurz A. (2024). Experiences of Young Adults Affected by Cancer Within an 8-Week Yoga Intervention Delivered by Videoconference: A Qualitative Interview Study. Ann. Med..

[37] Delgado-Guay M.O., Palma A., Duarte E., Grez M., Tupper L., Liu D.D., Bruera E. (2021). Association Between Spirituality, Religiosity, Spiritual Pain, Symptom Distress, and Quality of Life Among Latin American Patients with Advanced Cancer: A Multicenter Study. J. Palliat. Med..

[38] Ciria-Suarez L., Calderon C., Fernández Montes A., Antoñanzas M., Hernández R., Rogado J., Pacheo-Barcia V., Ansensio-Martínez E., Palacín-Lois M., Jimenez-Fonseca P. (2021). Optimism and Social Support as Contributing Factors to Spirituality in Cancer Patients. Support. Care Cancer.

[39] Hoseini L., Lotfi Kashani F., Akbari S., Akbari M.E., Sarafraz Mehr S. (2016). Model Development of Illness Perception and Consequences in Breast Cancer Patients. Asian Pac. J. Cancer Prev..

[40] Ahmadi N., Ahmadi F. (2017). The Use of Religious Coping Methods in a Secular Society. Illn. Crisis Loss.

[41] Kugbey N., Asante K.O., Meyer-Weitz A. (2020). Illness Perception and Coping among Women Living with Breast Cancer in Ghana: An Exploratory Qualitative Study. BMJ Open.

[42] Prince P., Mitchell S.A., Wehrlen L., Childs R., Savani B., Yang L., Bevans M. (2015). Spiritual Well-Being in Hispanic and Non-Hispanic Survivors of Allogeneic Hematopoietic Stem Cell Transplantation. J. Psychosoc. Oncol..

[43] Phenwan T., Peerawong T., Tulathamkij K. (2019). The Meaning of Spirituality and Spiritual Well-Being among Thai Breast Cancer Patients: A Qualitative Study. Indian J. Palliat. Care.

[44] Mohebbifar R., Pakpour A.H., Nahvijou A., Sadeghi A. (2015). Relationship Between Spiritual Health and Quality of Life in Patients with Cancer. Asian Pac. J. Cancer Prev..

[45] Moysés R., Marques I., Santos B.D., Benzaken A., Pereira M.G. (2023). Quality of Life in Amazonian Women During Cervical Cancer Treatment: The Moderating Role of Spirituality. Int. J. Environ. Res. Public Health.

[46] Mallaiah S., Narayanan S., Wagner R., Cohen C., Christie A.J., Bruera E., Lopez G., Cohen L. (2022). Yoga Therapy in Cancer Care via Telehealth During the COVID-19 Pandemic. Integr. Cancer Ther..

[47] Park S., Sado M., Fujisawa D., Sato Y., Takeuchi M., Ninomiya A., Takahashi M., Yoshimura K., Jinno H., Takeda Y. (2018). Mindfulness-Based Cognitive Therapy for Japanese Breast Cancer Patients—A Feasibility Study. Jpn. J. Clin. Oncol..

[48] Tan S.B., Chee C.H., Ngai C.F., Hii S.L., Tan Y.W., Ng C.G., Capelle D.P., Zainuddin S.I., Loh E.C., Lam C.L. (2023). Mindfulness-Based Supportive Therapy on Reducing Suffering in Patients with Advanced Cancer: Randomised Controlled Trial. BMJ Support. Palliat. Care.

[49] Oliveira S.S.W., Vasconcelos R.S., Amaral V.R.S., Sousa H.F.P.E., Dinis M.A.P., Vidal D.G., Sá K.N. (2021). Spirituality in Coping with Pain in Cancer Patients: A Cross-Sectional Study. Healthcare.

[50] Zetzl T., Renner A., Pittig A., Jentschke E., Roch C., van Oorschot B. (2021). Yoga Effectively Reduces Fatigue and Symptoms of Depression in Patients with Different Types of Cancer. Support. Care Cancer.

[51] Chang T.-G., Hung C.-C., Huang P.-C., Hsu C.-Y., Yen T.-T. (2022). Demoralization and Its Association with Quality of Life, Sleep Quality, Spiritual Interests, and Suicide Risk in Breast Cancer Inpatients: A Cross-Sectional Study. Int. J. Environ. Res. Public Health.

[52] Tsai T.-J., Chung U.-L., Chang C.-J., Wang H.-H. (2016). Influence of Religious Beliefs on the Health of Cancer Patients. Asian Pac. J. Cancer Prev..

[53] Tan T.T., Tan M.P., Lam C.L., Loh E.C., Capelle D.P., Zainuddin S.I., Ang B.T., Lim M.A., Lai N.Z., Tung Y.Z. (2023). Mindful Gratitude Journaling: Psychological Distress, Quality of Life and Suffering in Advanced Cancer: A Randomised Controlled Trial. BMJ Support. Palliat. Care.

[54] Velasco-Durantez V., Cruz-Castellanos P., Hernandez R., Rodriguez-Gonzalez A., Fernandez Montes A., Gallego A., Manzano-Fernandez A., Sorribes E., Zafra M., Carmona-Bayonas A. (2024). Prospective Study of Predictors for Anxiety, Depression, and Somatization in a Sample of 1807 Cancer Patients. Sci. Rep..

[55] Liu W., Liu J., Ma L., Chen J. (2022). Effect of Mindfulness Yoga on Anxiety and Depression in Early Breast Cancer Patients Received Adjuvant Chemotherapy: A Randomized Clinical Trial. J. Cancer Res. Clin. Oncol..

[56] Zhi W.I., Baser R.E., Zhi L.M., Talukder D., Li Q.S., Paul T., Patterson C., Piulson L., Seluzicki C., Galantino M.L. (2021). Yoga for Cancer Survivors with Chemotherapy-Induced Peripheral Neuropathy: Health-Related Quality of Life Outcomes. Cancer Med..

[57] Shi X., Wang F., Xue L., Gan Z., Wang Y., Wang Q., Luan X. (2023). Current Status and Influencing Factors of Spiritual Needs of Patients with Advanced Cancer: A Cross-Sectional Study. BMC Nurs..

[58] Sun X.-H., Liu X., Zhang B., Wang Y.-M., Fan L. (2021). Impact of Spiritual Care on the Spiritual and Mental Health and Quality of Life of Patients with Advanced Cancer. World J. Psychiatry.

[59] Guo Y.-Q., Ju Q.-M., You M., Yusuf A., Wu Y., Soon L.K. (2022). A Qualitative Study on Coping Strategies of Chinese Women with Metastatic Breast Cancer Undergoing Chemotherapy. Front. Psychol..

[60] Zamaniyan S., Bolhari J., Naziri G., Akrami M., Hosseini S. (2016). Effectiveness of Spiritual Group Therapy on Quality of Life and Spiritual Well-Being Among Patients with Breast Cancer. Iran J. Med. Sci..

[61] Bożek A., Nowak P.F., Blukacz M. (2020). The Relationship Between Spirituality, Health-Related Behavior, and Psychological Well-Being. Front. Psychol..

[62] Puchalski C.M. (2001). The Role of Spirituality in Health Care. Bayl. Univ. Med. Cent. Proc..

[63] Büssing A., Recchia D.R., Koenig H., Baumann K., Frick E. (2018). Factor Structure of the Spiritual Needs Questionnaire (SpNQ) in Persons with Chronic Diseases, Elderly and Healthy Individuals. Religions.

[64] Büssing A. (2021). The Spiritual Needs Questionnaire in Research and Clinical Application: A Summary of Findings. J. Relig. Health.

[65] Kamraju M. (2023). Yoga and Its Spirituality. ASEAN J. Community Engagem..

[66] Vallath N. (2010). Perspectives on Yoga Inputs in the Management of Chronic Pain. Indian J. Palliat. Care.

[67] Lazaridou A., Pentaris P. (2016). Mindfulness and Spirituality: Therapeutic Perspectives. Pers. Centered Exp. Psychother..

[68] Tarakeshwar N., Vanderwerker L.C., Paulk E., Pearce M.J., Kasl S.V., Prigerson H.G. (2006). Religious Coping Is Associated with the Quality of Life of Patients with Advanced Cancer. J. Palliat. Med..

[69] Henning M.A., Lyndon M., Ng L., Sundram F., Chen Y., Webster C.S. (2024). Mindfulness and Religiosity: Four Propositions to Advance a More Integrative Pedagogical Approach. Mindfulness.

[70] Salsman J.M., Fitchett G., Merluzzi T.V., Sherman A.C., Park C.L. (2015). Religion, Spirituality, and Health Outcomes in Cancer: A Case for a Meta-Analytic Investigation. Cancer.

[71] Koenig H.G. (2008). Concerns About Measuring “Spirituality” in Research. J. Nerv. Ment. Dis..

[72] Visser A., Garssen B., Vingerhoets A.J. (2017). Existential Well-Being: Spirituality or Well-Being?. J. Nerv. Ment. Dis..

[73] Sepúlveda C., Marlin A., Yoshida T., Ullrich A. (2002). Palliative Care: The World Health Organization’s Global Perspective. J. Pain Symptom Manag..

[74] Quinn B., Connolly M. (2023). Spirituality in Palliative Care. BMC Palliat. Care.

[75] Miller M., Addicott K., Rosa W.E. (2023). Spiritual Care as a Core Component of Palliative Nursing. Am. J. Nurs..

[76] Best M.C., Vivat B., Gijsberts M.-J. (2023). Spiritual Care in Palliative Care. Religions.

